# Animal Welfare Compromises Associated with Causes of Death in Neonatal Piglets

**DOI:** 10.3390/ani12212933

**Published:** 2022-10-26

**Authors:** Kirsty L. Chidgey, Nutnapong Udomteerasuwat, Patrick C. H. Morel, Fernanda Castillo-Alcala

**Affiliations:** 1 School of Agriculture and Environment, Massey University, Palmerston North 4474, New Zealand; 2 College of Veterinary Medicine, Michigan State University, East Lansing, MI 48824, USA; 3 Faculty of Veterinary Medicine and Applied Zoology, The HRH Princess Chulabhorn College of Medical Science, Bangkok 10210, Thailand; 4 Tāwharau Ora—School of Veterinary Science, Massey University, Palmerston North 4474, New Zealand

**Keywords:** piglet, mortality, post-mortem, five domains, welfare

## Abstract

**Simple Summary:**

The pre-weaning piglet mortality percentage is a commonly reported metric on commercial pig farms. The mortality percentage tells us how many piglets died, but not what their welfare status was as it relates to their cause of death. This pilot study aimed to evaluate the likely experience of piglets that died, following confirmation of the cause of death via postmortem investigation. The Five Domains Model was then used to collate scientific evidence of the likely experience of these piglets before death from acute disease, starvation, crushing, savaging and euthanasia, to understand the impact of different causes on their welfare. The resulting findings raised the question of differentiating ‘smothering’ as a cause of death from ‘crushing,’, and that co-morbidities (such as hypothermia) may alter the welfare experience due to their influence on consciousness before death.

**Abstract:**

This pilot study aimed to assess the welfare impacts of different causes of pre-weaning deaths in piglets. Piglets that died between 0–7 days after birth (*n* = 106) were collected from two commercial pig farms and subject to post-mortem examination to confirm their cause of death as well as any contributing factors. Using the Five Domains Model, the most likely affective experiences associated with the pathological findings were carefully inferred to better understand affective experience as it related to known causes of liveborn piglet mortality. The most common causes of liveborn piglet mortality were starvation (23%), crushing (23%) and non-viable (21%). Thirty one piglets had evidence of starvation, but it was only considered the primary cause of death in 15 piglets, as cofactors such as poor viability (*n* = 13) were also present in many piglets with evidence of starvation. All 15 piglets that were crushed died within 24 h after birth and most had evidence of thoracic and/or abdominal internal bleeding. This study found that common causes of liveborn piglet death were associated with compromises in Domains 1 (Nutrition/hydration), 3 (Health/function), and4 (Behavioural interactions), with the most likely resulting affective states described in Domain 5 (Mental state). This highlights the interaction between physical/functional and situation-related (behavioural) aspects that influence an animals’ welfare status.

## 1. Introduction

Pre-weaning piglet mortality (PWM) represents the percentage of piglets born alive that died before weaning. Whilst the loss of piglets does have a financial implication, emphasizing mortality rates as a key performance indicator and a matter of economics undermines the piglet welfare implications and a pig keeper’s commitment to animal welfare, as piglet deaths are expected to be associated with some welfare compromise in most instances. As an indicator, PWM conveys no information on the cause of death each piglet experienced, each of which potentially having different welfare implications. On its own PWM tells us nothing about the impact of these causes of death on welfare other than to perhaps assume that the impact goes up as PWM increases. Therefore, the exclusive use of mortality rate may limit our understanding towards pathophysiology and affective neuroscience in neonatal piglets in the context of welfare impacts. Previous studies investigating PWM have mainly focused on physiological factors [1] rather than the piglet’s affective (mental) experience despite an established relationship between an animal’s physiological status and their welfare status.

The Five Domains Model [2,3] is a tool that enables a structured and systematic assessment of animal welfare. It is not a means to define welfare in terms of a valence, i.e., ‘good’ vs. ‘bad’; but a framework to grade welfare compromise as well as welfare enhancement [4]. As outlined in Table 1, the Model is composed of two major categories: the physical/functional domains; and the affective experience domain. An animal’s affective state represents the culmination of inputs from the physical/functional domains. In the past, these elements were separately studied from each other, however, they are unified in recent literature [5]. This relationship between physical and mental states leads to a more comprehensive understanding of animal welfare [6,7]. The first three Domains: (1) Nutrition/hydration, (2) Physical environment and (3) Health/functional status, are related to the survival of the animal. The fourth Domain (4) Behavioural interactions, describes situation-related experiences which mainly focus on the perception of external circumstances and not survival-critical behaviours, e.g., hunting behaviours which are motivated by hunger [8]. Domain 4 is further split into three aspects relating to an animal’s interactions with their environment, other animals and with humans. The last Domain, (5) Mental state, is the affective experiences in Domains 1 to 4. Since the mental state is subjective, it cannot be evaluated directly. Therefore, indirect indicators have been applied to describe this experience-related state through physiological and behavioural responses [5]. Welfare status is then a summary of the affective experience of the animal [2]. Where possible, science-based evidence must accompany all assumptions about an animal’s affective state. Evidence may include direct observations of animal-based physical, physiological, clinical and behavioural data. Detailed records of PWM and causes of death are typically available on most commercial pig farms, given the widespread use of recording software (e.g., EliteHerd, PigCHAMP) to record and analyze performance metrics. The most common causes recorded are crushing, starvation and low viability behind which may be other contributing factors such as hypothermia and low birth weight [9,10,11,12]. Post-mortem examination is a valuable tool to confirm the cause of death and identify the trauma a piglet may have suffered to support an assessment of the potential animal welfare impacts that were present prior to or during death.

The objective of this study was to confirm the cause of death in piglets up to one week old by post-mortem examination and relate the findings to domain compromises and associated affective states using the Five Domains Model. The criteria to determine the cause of death for liveborn piglets included: crushing, starvation, euthanasia, acute disease, savaging, and low viability. Pathological findings were interpreted to determine potential antemortem experiences. The most likely affective experiences associated with these findings were carefully inferred to better understand affective experience as it relates to causes of mortality.

## 2. Materials and Methods

This was an observational study, whereby one hundred and six dead piglets (from <24 h–7 days old) were collected from two indoor pig farms in the North Island of New Zealand during April 2018 and February 2019. The interval of 0–7 days was chosen as the majority (80–90%) of piglet deaths occur in this period [13]. The recorded variables from 106 piglets (56 males, 47 females, and 3 with missing gender information) were analysed using descriptive statistics in Microsoft Excel 2016.

The sows and their piglets were housed in pens with farrowing crates. The sows were introduced to the farrowing accommodation from approximately 5 days before farrowing and restrained in a farrowing crate measuring approximately 2.1 m in length, with an adjustable width and fully slatted flooring. On both farms the farrowing rooms had fan-assisted ventilation to keep the room temperature between 20–22 °C. One farm provided infrared heat lamps for the first 5 days after farrowing, as well as a heated solid floor in the piglet creep area. The other farm utilized underfloor heating in the creep area and adjusted the temperature each week to meet the piglets’ thermal requirements.

The percentage of piglets in each category of death was calculated. The categories represented the ultimate cause of death as determined by post-mortem examination. The piglets included those that were found dead and those that were humanely euthanised, with the reason for euthanasia recorded. The causes of death were: Type I stillbirth (death before farrowing commenced), Type II stillbirth (death during farrowing), congenital abnormality, crushing, euthanasia, starvation, non-viable, acute disease, savaging, and unknown. Piglets were collected by farm staff in the morning and individually labelled with the following information: sow ID, suspected cause of death, date of birth, date of death and evidence of splay leg. Piglets were chilled at 3–5 °C until collection. Collection occurred between 0 and 24 h after euthanasia or death. Piglets were transported in insulated cooler bins and delivered to the Massey University post-mortem facility for examination.

Post-mortem examination occurred within 24 h of collection. All examinations were conducted by the same person. The cause of death classification system in this study was adapted from [1,14].

Each piglet was assigned to a primary cause of death category. This was not necessarily the ultimate cause of death. For instance, the ultimate cause of death of 15 piglets was humane destruction (euthanasia) although for 14 of these the primary cause necessitating this action was determined at post-mortem, e.g., the piglet was non-viable, had acute disease or a congenital abnormality. In some instances, more than one problem was identified at post-mortem, e.g., a piglet had injuries consistent with crushing and was also starved or non-viable. A hierarchy of cause was applied, although application of such a hierarchy is unlikely to be correct in every instance. For example, if a piglet at post-mortem was found to be non-viable and crushed, crushing was considered the primary cause of death. If a piglet was non-viable and starved, the piglet was classified as non-viable. Piglets that had non-aerated lungs were classified as either Type I or Type II stillbirths. Causes of death in liveborn piglets were: congenital abnormality, crushing, euthanasia, starvation, non-viable, acute disease, savaging, and unknown. Crushing was treated as either a secondary cause of death or a primary cause of death, based on the associated pathological findings. The PWM was not calculated as piglets were not collected from birth to weaning. Furthermore, a bias occurred as some causes of death (e.g., infectious disease) were either more or less likely to be present in the sampled population because of their age range.

The Five Domains Model [4] was used to systematically evaluate animal welfare compromise in relation to each known cause of death in liveborn piglets. The post-mortem data was the basis for evaluating the potential affects associated with causes of mortality. Domains 1–4 (Table 1) were evaluated first. Following this assessment, the affects were carefully assigned to Domain 5.

## 3. Results

### 3.1. Causes of Death

The cause of death of 106 piglets (56 males, 47 females, and 3 with missing gender information) was determined. Forty-one piglets (39%) out of 106 were stillborn, either Type I or Type II (Table 2). All piglets classified as stillborn had deciduous hoof capsules (eponychium), however, ten out of 50 piglets (20%) with eponychium were found to have died from other causes according to post-mortem examination. Average body weights of the Type I and Type II stillbirths were 1.57 ± 0.59 kg and 1.43 ± 0.54 kg respectively.

Amongst the 106 piglets, two piglets were classified as starved and had crushing injuries, five piglets were classified as non-viable and had crushing injuries. The cause of these deaths is recorded as crushing in Table 2. Two piglets were both starved and splay-legged. These deaths were recorded as starvation. Most of the non-viable piglets were also starved but were classified as non-viable. One of the two savaged piglets was also starved. The non-viable piglets (*n* = 14, 21%) had the lowest average weight (0.59 ± 0.55 kg). No attempt was made to demonstrate that this weight was significantly lower than that of other dead piglets as low weight was a component of the classification. Piglets that died due to acute disease had the highest average weight (1.96 ± 0.56 kg).

### 3.2. Pathological Causes of Death

#### 3.2.1. Stillbirth

All 41 stillborn piglets presented with wet, heavy, uninflated lungs on examination. The lung tissue sank in water confirming they had not breathed before they died. Meconium staining on the skin was observed on 27 piglets in total (25%), five of which (18%) were classified as type I stillborn and 11 (41%) as type II. Ten piglets (9%) had meconium in the trachea, whereby seven of these piglets also had meconium on the skin.

#### 3.2.2. Crushing

All 15 deaths due to crushing occurred within 24 h after birth. Five of the crushed piglets weighed under 800 g and therefore were also non-viable. Starvation was evident in two crushed piglets, as these had evidence of emaciation and dehydration which may have contributed to their deaths. Most of the crushed piglets (*n* = 15, 23%) had evidence of thoracic and/or abdominal internal bleeding. Five of these showed signs of bleeding caused by liver rupture, however the site of bleeding could not be identified in the remaining piglets (Table 3). Ten crushed piglets showed skin and/or subcutaneous lesions (e.g., bruising) on the body. Six piglets had evidence of a skull fracture (Table 3). Only one piglet had intracranial damage and this piglet also had blood in the stomach. None of the crushed piglets had rib fractures. Nine crushed piglets had evidence of milk curds in the stomach, whereas the stomachs of the remaining six pigs were empty at the time of death.

#### 3.2.3. Euthanasia

Fifteen of the 65 liveborn piglets (23%) had been euthanised on farm using blunt force trauma (Table 2). The reasons for euthanasia were recorded by farm staff and pathological findings were recorded in euthanised piglets. Five piglets had clinical signs of disease, two had congenital abnormalities, three were non-viable, four had evidence of starvation and one was unknown. All euthanised piglets had evidence of skull fractures (Table 3). Twelve piglets had variable degrees of brain parenchyma damage, with focally extensive hemorrhages, while the remaining three had subdural hematomas. Different types of fractures were presented (comminuted, depressed and suture line). Three piglets had evidence of internal bleeding in the thorax and/or abdomen. Hemoabdomen in two of these three piglets was attributed to liver rupture.

#### 3.2.4. Starvation

Of 31 piglets with evidence of starvation, it was only considered the primary cause of death in 15 (23%). Thirteen piglets with evidence of starvation were classified as non-viable, two were attributed to crushing and one to savaging (Table 2). None of these piglets were found to have milk in the stomach, which was the main criterion used in this classification. Ten of the 15 piglets where starvation was the considered the primary cause of death died within 24 h of birth. The average body weight was 1.40 ± 0.54 kg. Two piglets classified as having starved had evidence of splay leg which was confirmed by on-farm diagnosis.

#### 3.2.5. Savaging, Acute Disease, and Non-Viable Piglets

Two piglets were classified as savaged with external laceration, skull fracture, massive hemorrhage and blood in the stomach being found upon post-mortem examination. Seven piglets (7%) out of 106 were determined to have died because of acute disease (Table 2). In the present study, the acute disease syndromes identified included enteritis, arthritis, and respiratory disease. All but one piglet had enlarged lymph nodes, one piglet had a severe pneumonia, and another had fibrinous pleuritis. Nineteen piglets (18%) weighed under 800 g and were classified as non-viable (Table 2). For five of these, crushing was considered the primary cause of death. Evidence of having ingested milk was present in only three non-viable piglets.

The cause of death could not be identified in ten piglets (9%) (Table 2), which had no obvious lesions, co-factors or underlying causes associated with mortality. All these piglets had evidence of suckling as milk curds were present in the stomach.

### 3.3. Evaluation of Welfare Compromise

The known causes of liveborn piglet death attributed to starvation, acute disease, savaging, and crushing were evaluated against the Five Domains Model [4] (Table 4). Some 15 pigs were euthanized using blunt force trauma and all suffered significant physical trauma during the process as is evident in Table 3, i.e., all 15 piglets had skull fractures. The staff on the two farms from which the piglets were obtained were trained to perform euthanasia efficiently and in a manner that renders the piglet insensitive instantaneously. Therefore, no suffering other than that associated with the condition that precipitated the decision to euthanize the animal was considered relevant. However, there may be an argument for considering impacts in Domain 4 attributed to the human handling just prior to, and during, euthanasia.

Non-viable piglets were not considered separately in this evaluation as they were represented among piglets that died from starvation and crushing. The pathological evidence was used to infer the likely affective states associated with each mortality cause.

In the present study, eleven piglets were found to have internal bleeding associated with being crushed, while a lack of any gross fatal lesions accompanying crushed piglets led to a suspicion of smothering as opposed to crushing. Death by smothering may occur whilst a piglet is not fully conscious, in which case there may be a reduced risk of welfare compromise. However, if these piglets were conscious for at least part of the event, compromised animal welfare is likely. Piglets that retain some level of consciousness during crushing or smothering may experience compromises relating to domains 3 (health), 4 (behavioural interactions) and 5 (mental state) (Table 4). Injury and functional impairment would result in a compromise to Domain 3. Under these circumstances, the piglets would be unable to avoid or escape the life-threatening behaviour from the sow, therefore crushing (and smothering) is also likely to be associated with a compromise in Domain 4 (behavioural interactions). Negative affective states experienced are likely to be pain, fear, panic, dizziness, breathlessness, and helplessness, as described in Domain 5.

At least 15 piglets died due to starvation (many non-viable piglets were also starving). Restriction of feed intake is consistent with a compromise in two of the survival-related domains: Nutrition/hydration (Domain 1) and health (Domain 3). Absence of milk in the stomach could arise from factor(s) that inherently reduce the ability of the piglets to suckle (e.g., disease, weakness), reduce the availability of milk (e.g., mastitis), or access to milk (e.g., teat competition). Given the latter there is potential that Domain 4 (behavioural interactions) may also be involved. The likely negative affective experiences described in Domain 5 associated with starvation would be hunger, thirst, weakness, dizziness, and frustration.

Seven piglets were affected by acute disease. Post-mortem examination found evidence of arthritis, enteritis and respiratory disease. All piglets with disease probably experienced a Domain 3 compromise associated with clinical symptoms. Those with respiratory disease may have experienced functional impairment of the respiratory system with the potential affective experiences including pain, breathlessness, and lethargy. Those with evidence of enteritis and dehydration potentially experienced negative affects in association with Domain 1.

Two piglets were killed due to savaging and had lesions associated with trauma. These represent a compromise in animal welfare assuming the piglet was conscious at the time, which is consistent with Domain 3 (health/function), and Domain 4 (behavioural interactions) compromise. Similarly for crushing, the piglet was unable to avoid the sow’s savaging behaviour which probably resulted in the mental experiences of fear and panic. Additional affective experiences associated with acute injury are likely to be pain, anxiety, and helplessness.

Although it was not clear what the ultimate cause of death was in piglets classified as ‘unknown’, it was hypothesized that they may have died from asphyxia given minimal, or a lack of, gross lesions. One reason for this hypothesis was the observation from stockpersons on one farm that sometimes piglets are smothered, rather than crushed. This was explained as occurring when a piglet falls asleep at the sow’s udder and the sow moves subtly, occluding the mouth and nasal passages of the piglet, causing suffocation. Another reason for this potential re-categorization was that all the piglets in the ‘unknown’ category were recorded by staff as having been crushed by the sow. As a result of asphyxia, animal welfare compromise could involve domains 3, 4 and 5 with similar experiences as described for crushing. If the piglets were asleep, they may not have become aware before their death, thus there is the possibility that they did not experience any welfare compromise. However, there is evidence that hypercapnia directly activates midbrain neurons that act as lifesaving “arousal chemoreceptors” to promote wakefulness in response to increasing CO_2_ levels [15].

## 4. Discussion

Sentience and consciousness are necessary preconditions of suffering and, therefore, in assessing domain-compromised animal welfare. Without sentience, an animal is unable to feel and without consciousness an animal cannot suffer. Based on a three-category system classifying neurological maturity at birth, piglets were placed in the most mature category due to their comparatively advanced neurological and behavioural development [16]. Farm animals, including piglets, are considered to be sentient shortly before birth due to their neurological development and may be considered conscious within minutes of birth [17]. This is achieved through the very rapid reduction in circulatory and cerebral adenosine concentrations through the loss of the placental source of adenosine and hyperoxaemia as the animal begins to breathe [17]. It is presumed, therefore, that all of the piglets that had aerated lungs had gained consciousness. However, a limitation of this study was that no information was available to determine the extent to which piglets remained conscious during or after the adverse event(s) that led to their subsequent deaths, i.e., when, or if, during a crushing event a piglet lost consciousness prior to its death was unknown.

Post-mortem examination can describe the antemortem changes that occurred in a piglet, and while these may imply it experienced pain and other noxious stimuli, post-mortem cannot determine how conscious the animal was immediately at, or following, exposure to noxious stimuli. Following some noxious stimuli, such as crushing, the piglet may either be conscious or unconscious during the period before death and the length of this period would vary also between piglets. This will impact on the amount of suffering experienced. Piglets that died due to starvation are expected to have been conscious for most of this period. Conscious piglets could experience the affective state(s) associated with different noxious events for minutes or hours. Moreover, it can be far more complicated where dull consciousness occurs in certain situations (i.e., hypothermia, breathlessness) [18]. In this study, we have assumed that if piglets were conscious immediately after a noxious stimulus, they would be able to experience the related affective state(s).

The post-mortem technique used in this study may or may not have underestimated the actual number of piglets that died due to the identified causes. However, no single method of investigation could determine such information. Video recording for example may provide confirmation of some causes of death, particularly in crushing or injury-related scenarios [19,20,21]. However, other causes (e.g., hypothermia, starvation, specific disease) may not be determined from observation alone.

Furthermore, the accuracy with which the primary cause of death could be determined was limited. For instance, where a piglet was found to be both starved and crushed, death was attributed to crushing. While it is probably reasonable to consider crushing as the ultimate cause of death, that crushing occurred may be due to a hypoglycaemic state, itself secondary to starvation which may influence our understanding of why some piglets died. However, it does not alter the reality that the crushed piglet had, by definition, suffered internal trauma (Table 3) and that this would have had implications for welfare status antemortem.

Whilst the piglets in this study were sourced from one type of farrowing system (pens with farrowing crates), the implications of this study are that the welfare impacts experienced by piglets may vary between different farrowing system designs and be greater in those where piglets are more vulnerable to chilling, crushing and starvation. Individual mortalities are inevitable and to some extent, unavoidable in all types of commercially used farrowing systems (e.g., farrowing crates, farrowing pens, and farrowing huts outdoors). However, it is known that PWM differs between these different systems. For instance, it is higher in outdoor huts (e.g., 21%, [22]) and freedom farrowing pens (e.g., 18–20%, [23,24,25] than in farrowing pens with temporary crating (e.g., 14–17%, [26,27,28] or pens with conventional farrowing crates (e.g., 10–12%), [29,30]. The distribution of causes of death also varies between systems, with crushing more common than other causes in outdoor and loose pen-based systems compared to farrowing crates [31,32]. Given the main causes of liveborn mortality and their interrelatedness, e.g., non-viable piglets are predisposed to hypothermia/starvation, and therefore crushing, while hypothermic piglets are also less viable; affective states such as breathlessness, pain, and hunger are likely to be experienced by many piglets before death. Somewhat paradoxically, some co-factors (e.g., hypothermia, disease) may inhibit the conscious awareness of noxious events whereas piglets that are otherwise healthy and fully aware may experience more significant compromise.

The most common causes of piglet mortality in this study were starvation (23%), crushing (23%) and low viability (21%). Another study that performed postmortem examinations of 798 piglets that died within 5 days of birth reported similar results to these, where the three most common causes of death were starvation (34%), crushing (28%) and enteritis (24%) [25]. An analysis of records from 2143 litters reported that crushing of healthy piglets was the most common cause of liveborn preweaning mortality (54.8%) followed by low viability (13.8%), starvation (6.8%) and unknown (6.1%) [13]. It is possible that crushing in the present study was under diagnosed due to a lack of obvious lesions in some piglets. Some of the piglets for which no cause was established could have been asphyxiated without evidence of injury, as was speculated by stockpersons who recounted finding otherwise healthy, apparently smothered piglets in the past. Accompanying information such as the suspected cause of death is an important detail to assist with diagnosing mortality causes post-mortem, however there can still be some incongruence between this information and pathological evidence. Video footage has been used to confirm the cause of death when post-mortem evidence was inconclusive, or the reason given by farm staff was not supported by post-mortem evidence [33]. One potential influence in the present study was the elapsed time between death and the refrigerated storage of piglets on-farm, which may also have affected the accuracy of some diagnoses.

### Domain Compromise and Affective States

Starvation as a cause of death in neonatal piglets is often coincidental with hypothermia, particularly when piglets die within the first 24 h of birth. Piglets are particularly susceptible to hypothermia in early life as they are virtually hairless at birth, with low energy reserves and a lack of brown adipose tissue [34]. Shivering is the principal mechanism of thermoregulation, which in itself is energetically expensive and can compromise muscular function [11]. At birth, the piglet’s lower critical temperature is approximately 34 °C whereas the recommended temperature in a farrowing environment for lactating sows is 18–21 °C [35]. Therefore, if the temperature is comfortable for the sow, it may lead to environmental challenges for the piglets such as decreased colostrum consumption leading to energy depletion, weakness, starvation, and an inability to avoid the sow’s risky movements [36,37]. The design of the farrowing environment is an important factor in preventing such thermal challenges for piglets. Common features to avoid chilling and hypothermia include the provision of piglet-only ‘creep’ areas, which may feature a cover, a heated floor and/or infrared heat lamps; and/or bedding such as straw.

Starvation was classified by a lack of milk in the stomach, and evidence of emaciation and dehydration. Immediately after birth piglets must be able to access colostrum because it plays such a crucial role in promoting their health and survival. For this reason, cross fostering (transferring piglets to equalize litter sizes within 12–24 h after farrowing) is undertaken on commercial farms when necessary to ensure all piglets have access to a functional teat, thereby preventing starvation and reducing piglet mortality [38]. Colostrum is a source of readily digestible nutrients, immunoglobulins, growth factors, enzymes and hormones, all contributing to the development of the immune system, thermoregulation, and gut maturation [39,40]. The amount of colostrum consumed, and the amount of glycogen stores present are vital as both are essential for energy metabolism [41]. Several studies show a relationship between energy metabolism and thermoregulation in neonatal piglets. The rectal temperature of piglets at 24 h post birth correlates positively with colostrum ingestion [42,43]. Piglets deprived of colostrum that experienced ambient temperatures of 34 °C could not maintain their body temperature and began to experience cold stress resulting in increased use of their energy reserves through gluconeogenesis [35]. The likely affective experience associated with hypothermia will depend on its severity (mild, moderate, or severe), duration (minutes to hours) and whether the severity is of a magnitude that leads to depressed cognitive function [18]. As hypothermia progresses in piglets that are aware, they may endure negative mental states including thermal discomfort, lethargy, exhaustion, and confusion. Conscious piglets that starve to death will experience severe hunger. If this is accompanied by hypothermia, the experience may not be continuous given a reduced level of consciousness as both conditions progress [18]. Without the impedance of hypothermia, however, piglets would likely experience hunger, thirst, dizziness, and weakness due to hypoglycemia as the effects of starvation advance.

In a pig herd the incidence and causes of disease will vary as a function of factors such as management, hygiene, nutrition, herd health status, preventative health measures and genetics. In the present study, the acute disease syndromes identified included enteritis, arthritis and respiratory disease. If not immediately fatal, wounds or injuries can be a means of introducing bacteria to the piglets’ systemic circulation [44]. Such wounds can be the cause of infectious arthritis in piglets under four days of age [45]. Functional impairment may result with advancing infection, and in the case of the two piglets that had evidence of respiratory disease, it was considered probable that they would have experienced breathlessness, air hunger and dizziness before loss of consciousness and euthanasia or death. Depending on its severity, an infection is likely to be associated with suffering including experiences of pain, weakness, and lethargy; although the latter is typically the consequence of sleepiness or drowsiness, which is commonly observed in sick animals and may mitigate the experience of negative affects, albeit temporarily [20].

Birth weight is an important predictor of postnatal survival [42,46]. Piglets with a birth weight of 700 g had a survival rate of only 33% [47], vs. a 68% and 89% survival rate in piglets weighing 900 g or 1.6 kg at birth, respectively [48]. In the present study, piglets less than 800 g were classified as non-viable. Five non-viable piglets were crushed by their dam. In agreement with previous literature, low viability was in some cases a conjunctive cause of death rather than the ultimate cause of death. Non-viable piglets have reduced energy reserves at birth which compromises thermoregulation, making them more susceptible to hypothermia, starvation, and crushing [49,50].

An important consideration when assessing the welfare impact of crushing is whether piglets were conscious during the event, as dull consciousness reduces the noxious effect of a negative affective experience [17,18]. Whilst crushing as a cause of death may be confounded by other physical and/or physiological compromises including low viability, starvation, hypothermia, and lack of oxygen during the birth process (perinatal hypoxia), [49,50,51] a previous analysis of piglet deaths found that 54% of liveborn mortality was attributed to the crushing of healthy piglets [13]. This finding is similar to the present study where half of those classified as crushed had no obvious co-morbidities. Thus, it is possible that at least half of the piglets were fully conscious when they were crushed. A study examining crushing behaviour of 24 sows and the subsequent survival of overlain piglets found 95% of piglets survived being crushed for <1 min, but only a third survived being crushed for >4 min [49]. Piglet distress calls are correlated with crushing events to the extent that the ‘piglet scream test’ has been used in many previous experiments to evaluate a sow’s maternal responsiveness by simulating an overlaying event [19,21,52]. Only conscious piglets would be capable of emitting a distress vocalisation during crushing to induce a reactive response from the sow. The use of a piglet vocalisation cue to simulate a sow’s response to a crushing situation suggests that it is highly likely piglets are conscious for at least some period during crushing events. As such they are likely to have a negative affective experience. For the piglets that die after being crushed for a prolonged period, it is suggested the mechanism is asphyxia and subsequent reduction of blood oxygen levels. A review of fatal asphyxia episodes in humans described some mechanisms involved [53]. These include: external airway obstruction or smothering (occluding the external air passages, e.g., nose or mouth); extrinsic compromise of thoracic cage function (body wall compression restricts movement of the respiratory muscles, e.g., the intercostal muscles and the diaphragm, reducing lung capacity during inspiration): and intrinsic compromise of thoracic cage function (e.g., multiple rib fractures and tension pneumothorax) [53].

Based on the above, during a crushing event, external airway obstruction and extrinsic compromise of thoracic cage function are the most likely explanation underlying death by asphyxia. The pathological findings in this study identified thoracic and abdominal bleeding and liver rupture in piglets that had been crushed, which may be evidence of compromised thoracic cage function. Significant blood loss resulting from internal bleeding can lead to circulatory disturbances, poor tissue perfusion, cellular hypoxia, tissue damage, organ dysfunction, and eventually death by shock [54]. Human patients that experienced hypovolemia reported feeling anxious, confused, dizzy, and lethargic. Thus, negative affective experiences are likely to be associated with moderate to severe blood loss in piglets that remined conscious for a period following the associated injury that caused blood loss.

Breathlessness describes a subjective state representing a negative affective experience. Breathlessness may be associated with visible outward signs such as dyspnea or labored breathing, which cannot clearly explain mental states, especially in animals [55]. Elements of breathlessness experienced by overlain piglets may also include air hunger and respiratory effort where thoracic movement is restricted [56]. In summary, the likely affective states experienced during crushing would be pain, fear, panic, dizziness, breathlessness, and helplessness.

The most important objectives when performing animal euthanasia are the achievement of rapid loss of consciousness, and the avoidance of stress and pain [57]. As mentioned, the influence of human handling just prior to and during euthanasia may warrant consideration in terms of impact in Domain 4. The New Zealand Code of Welfare for Pigs (2018) [58] includes a minimum standard for euthanasia. Emergency humane destruction of piglets must immediately result in loss of consciousness that persists until death. Manual blunt force trauma achieves these requirements and is currently an accepted method of emergency humane killing. Blunt force trauma applied to the cranium of the piglet produces structural brain damage, hemorrhage, and skull fractures. Brain damage is mainly found in the frontal lobe(s), with the most severe damage being to the occipital lobe(s) [59]. Based on an understanding that sentience and consciousness are preconditions of suffering, it is unlikely that appropriate euthanasia techniques will lead to an animal suffering [17]. However, an unsuccessful euthanasia technique may cause pain, dizziness, nausea, breathlessness, and panic prior to loss of consciousness.

## 5. Conclusions

In this study, data collected during post-mortem examination of piglets provided the scientific evidence to assess the potential animal welfare impacts of different causes of death. Using the Five Domains Model, common causes of liveborn piglet death were associated with compromises in Domains (1), (3), (4), and the related affective states described in Domain (5). This highlights the interaction between physical/functional and situation-related (behavioural) aspects that influence an animals’ welfare status. Crushing, starvation and low viability are common causes of death in piglets pre-weaning and can be co-occurring contributors to mortality. An improved understanding of the etiology of crushing and smothering warrants further investigation, as the most serious animal welfare impacts are likely to be experienced by fully conscious piglets that experience noxious stimuli for a period before death.

## Figures and Tables

**Table 1:** Components of The Five Domains Model (modified from [2]).

Domain			Valences	Example
1. Nutrition and hydration	Survival-related factors	Physical or functional domains	Positive	Eat enough food, eat a balanced diet, eating a variety of foods
			Negative	Restricted food intake, force-feeding
2. Physical environment			Positive	Suitable substrate, space for freer movement
			Negative	Thermal extremes, close confinement, unpredictable events
3. Health/function			Positive	Little or no disease, injury, functional impairment, appropriate body condition, good fitness level
			Negative	Presence of disease (acute or chronic), injury (acute or chronic), functional impairment due to lung, heart, kidney or other problems, obesity or leanness
4. Behavioural interactions	Situation-related factor		Positive	Free movement, playing, sexual activity, rest, exploration, bonding with human handlers
			Negative	Limits on threat avoidance, escape or defensive activity, constraints on environment-focused activity, uncertainty near humans
5. Mental state	Affective experience domain		Positive	Postprandial satiety, thermal comfort, comfort of good health and high functional capacity, calmness, energized
			Negative	Thirst, hunger, physical discomfort, thermal discomfort, pain, breathlessness, sickness, depression, anxiety, fearfulness, panic, physical exhaustion

**Table 2:** Overview of the number and percentage of piglets that died due to different causes as determined by post-mortem.

Cause of Death	*n*	% of Liveborn	Average Body Weight (kg) ± SD	No. Euthanised
Starvation	15	23	1.40 ± 0.54	4
Crushing	15	23	1.16 ± 0.56	0
Non-viable	14	21	0.59 ± 0.55	3
Unknown	10	15	1.45 ± 0.55	1
Disease	7	11	1.96 ± 0.56	5
Congenital abnormality	2	3	1.25 ± 0.55	2
Savaged	2	3	1.1	0
Type I Stillbirth	8		1.57 ± 0.59	0
Type II Stillbirth	33		1.43 ± 0.54	0
Total	106	100	1.32 ± 0.54	15

**Table 3:** Specific pathological findings associated with euthanasia and crushing.

Pathological Finding	Crushed (*n* = 15)	Euthanised (*n* = 15)
Petechiation	0	1
Bruising	10	8
Thoracic internal bleeding	2	1
Abdominal internal bleeding	11	3
Liver rupture	5	2
Skull fracture	6	15
Protruding tongue	15	8

**Table 4:** Summary of causes of death and the number of piglets experiencing Domain compromises. The likely affective states are described for each cause in Domain 5.

		Domains				
Cause of Death	Number of Piglets	1	2	3	4	5
Acute disease	7	2		7		Breathlessness, pain, weakness, dizziness, hunger, dehydration and lethargy.
Savaged	2			2	2	Fear, panic, pain, anxiety, helplessness.
Starvation	15	15		15		Hunger, thirst, weakness, lethargy, and dizziness. Potential for thermal discomfort if hypothermia is coincidental.
Euthanasia ^1^	15			15	15	Pain, fear, panic, uncertainty.
Crushing	15	2		15	15	Pain, fear, panic, dizziness, hunger, breathlessness and helplessness.

^1^ It is assumed that euthanasia was carried out correctly however there may have been a transitory negative experience associated with human handling prior to loss of consciousness and death.

## Data Availability

Contact the corresponding author.
